# Revealing the genetic mechanisms underpinning the parasite-induced water-seeking behaviour of insects through RNA-seq

**DOI:** 10.1098/rspb.2026.1414

**Published:** 2026-09-16

**Authors:** Upendra R. Bhattarai, Jean-François Doherty, Robert Poulin, Eddy Dowle, Neil J. Gemmell

**Affiliations:** ^1^ Center for Translational Neuroscience, Brown University, Providence, RI 02912, USA; ^2^ Department of Zoology, The University of British Columbia, Vancouver, British Columbia, Canada, V6T 1Z4; ^3^ Michael Smith Laboratories, Department of Biochemistry & Molecular Biology, The University of British Columbia, Vancouver, British Columbia, V6T 1Z4, Canada; ^4^ Department of Zoology, University of Otago, Dunedin 9016, New Zealand; ^5^ Department of Anatomy, University of Otago, Dunedin 9016, New Zealand; ^6^ New Zealand Institute for Bioeconomy Science Limited, Lincoln 7608, New Zealand

**Keywords:** host manipulation, water-seeking behaviour, dual RNA-seq, *Forficula auricularia*, *Mermis nigrescens*

## Abstract

Water-seeking behaviour in arthropods infected by mermithid nematodes or nematomorphs is a classic example of host manipulation, yet its molecular basis remains poorly understood. We investigated mechanisms underlying this aberrant behaviour in European earwigs (*Forficula auricularia*) infected with the nematode *Mermis nigrescens* using comparative RNA-seq across stages of infection and behavioural manipulation in both host and parasite. We detected 12 876 and 9722 expressed genes in earwigs and nematodes, respectively. Differential expression analyses identified 673 upregulated and 593 downregulated genes in earwigs, and 2672 upregulated and 2293 downregulated genes in nematodes across comparisons of infection stage and parasite size. Temporal clustering revealed six earwig and four nematode gene clusters. Gene ontology enrichment indicated increased signalling and sensory pathways in the host, while the parasite upregulated transport and secretory processes during manipulation, suggesting complementary and coordinated transcriptional changes between parasite and host. Notably, a shared GATA transcription factor motif was enriched among overexpressed genes in both organisms during manipulation. Together, these results identify candidate pathways and regulatory elements in host and parasite, providing new insights into the molecular regulation of parasite-induced water-seeking behaviour.

## 1. Introduction

Animals continuously make behavioural choices impacting their fitness and reproductive success. Contrary to the assumption that behaviours are self-determined and naturally selected for the animal's own fitness gain [1,2], instances exist where this is not the case [3–5]. Parasites often manipulate host behaviour to confer advantages upon themselves [6,7], a strategy that has independently evolved across all major parasite phylogenetic lineages [8]. Such manipulated hosts essentially serve as extended phenotypes of the parasites [9–11].

The water-seeking behaviour in arthropod hosts infected by parasitic worms (nematodes and nematomorphs) illustrates this phenomenon [12,13]. The evolutionary significance of this aberrant behaviour is particularly striking because of the apparent convergent evolution of this behaviour in parasites from two different phyla [14,15]. Nematomorphs and mermithid nematodes must emerge from their terrestrial arthropod hosts into water or water-saturated substrates to avoid desiccation, reproduce and thus complete their life cycle [16].

Despite historical documentation dating back over 140 years [17], the underlying mechanisms of parasite-induced water-seeking remain underexplored. Early proteomic investigations highlighted significant parasite–host molecular crosstalk during behavioural modification, suggesting possible biochemical pathways involved in host manipulation [18]. Research by Thomas and colleagues [19] identified neurotransmitter and neuromodulator alterations in the brain of the cricket *Nemobius sylvestris* infected by the nematomorph *Paragordius tricuspidatus*. The authors [20,21] carried out proteomic studies in grasshoppers and crickets infected with the nematomorphs *Spinochordodes tellinii* and *P. tricuspidatus*, respectively, correlating proteins from the Wnt signalling pathway with behavioural change. Moreover, although dehydration superficially increases interactions with water, protein profiling does not support this as a driving mechanism by hairworms [22].

Even less is known about the mechanisms underpinning the similar behavioural manipulation induced by mermithid nematodes. Williams *et al.* [23] found that the mermithid nematode *Thaumamermis zealandica* causes its amphipod host *Talorchestia quoyana* to burrow deeper into sand, down to water-saturated layers, following an increase in haemolymph osmolality after infection, suggesting some thirst-driven mechanism facilitating nematode emergence, yet this observation lacks a genetic explanation.

Transcriptomics offers insights into gene expression and physiological mechanisms dictating behaviour [24]. While high-throughput sequencing has advanced host–parasite interaction studies [25], the lack of comprehensive genomic data limits its application in non-model organisms. Dual RNA-seq, which profiles gene expression of both host and parasite, has been a popular approach [26–28]; however, this strategy is often complicated by RNA quality disparities between the host and the parasite when extracted together [29]. To address these issues, we adjusted our protocols to process host and parasite RNA separately, facilitating accurate dissection and individual sequencing. We also used a developmental series of both the parasite and the corresponding host. The use of a developmental time series across infections is crucial to differentiate between gene expression changes associated with infection and those associated with host manipulation. This research uses the European earwig (*Forficula auricularia*) and its parasitic mermithid nematode (*Mermis nigrescens*) to explore gene expression across different infection stages, aiming to elucidate the genetic mechanisms driving the observed water-seeking behaviour. Behavioural changes in earwigs occur primarily when mermithids have reached maturity [30]. As many cases of host manipulation in insects involve the central nervous system (CNS) [31], we hypothesize that substantial changes in the transcriptome should occur there. When their development within the earwig host is complete, these nematodes induce their host to seek water and enter it [30], allowing the worm to emerge where it needs to be to reproduce. Although some proteomic changes in earwig brains following infection by mermithid nematodes have been identified [14], these only provide a partial window into the underlying mechanisms responsible for host manipulation. Our comprehensive genomic approach focusing on both the host and parasite across developmental stages aims to address this knowledge gap.

## 2. Material and methods

### (a) Insect rearing

Adult earwigs (*F. auricularia*) were collected from the Dunedin Botanic Garden (−45°51′27.59″ S, 170°31′15.56″ E) and reared in a temperature-controlled room (temperature: cycling from 15 to 12°C, day/night; photoperiod of L : D 16 : 8) in the Department of Zoology, University of Otago, Dunedin, New Zealand. Earwigs were placed in a 20 l glass container with a dirt bed, dahlia flower petals and pieces of cardboard cuttings as cover. They were provided with food containing a 50 : 50 mixture of ground oats and commercial cat food ad libitum. Water was sprayed every second day to maintain moisture in the container. Earwigs were left for at least a week to acclimatize before sampling.

### (b) Sampling

**Figure 1.**
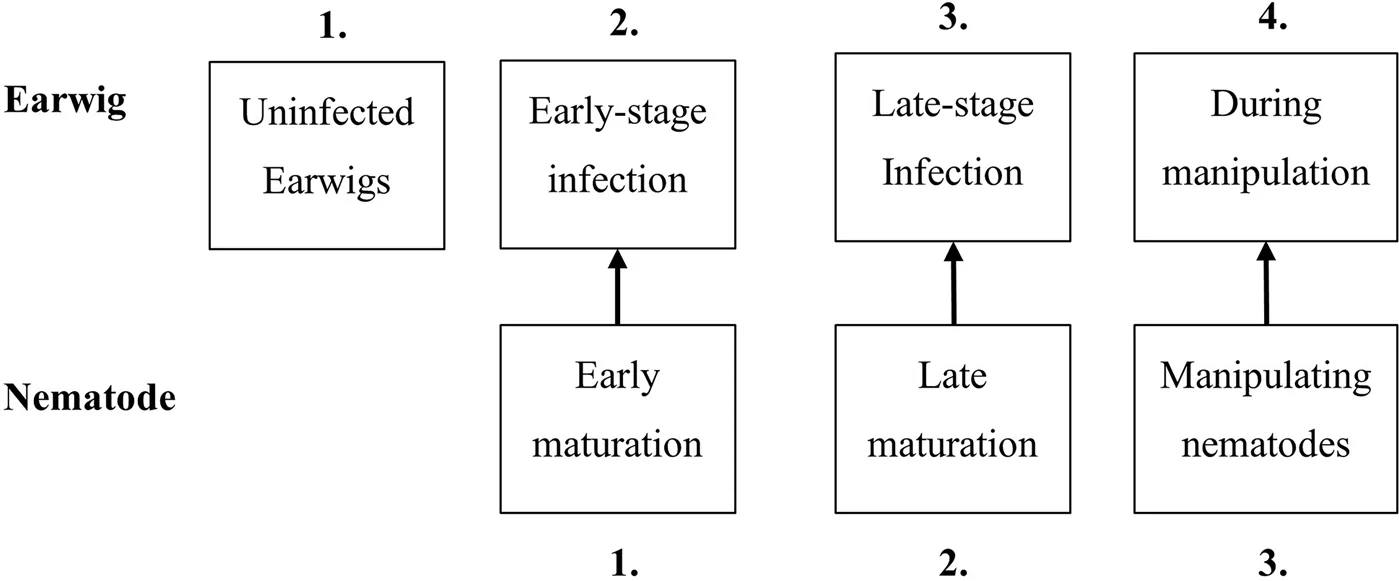
Sampling stages for RNA-seq studies of water-seeking behaviour. Earwigs were sampled at four time points: uninfected earwigs (UI), early-stage infection (EI), late-stage infection (LI) and during manipulation (DM), whereas nematodes were sampled at three time points: small nematodes (SN) extracted from earwigs at early stage of infection, large nematodes (LN) extracted from earwigs at late stage of infection, and manipulating nematodes (MN) extracted from earwigs during manipulation.

Naturally infected and uninfected (UI) earwigs were sampled across four time points (UI, early infection (EI), late infection (LI) and during manipulation (DM)) and nematodes across three time points (early maturation, late maturation and DM) (figure 1). Out of all individuals maintained as described above, all earwigs the behaviour of which was manipulated, that is those that entered water, were included in our analysis along with their nematode; for other stages, we used the first earwigs and nematodes we dissected that fell into these categories until we had matching numbers (see figure 1). Adult earwigs were snap-frozen in liquid N_2_ and dissected in 1× phosphate-buffered saline (PBS) under a dissection microscope to confirm the state and stage of parasitism. Samples were dissected quickly on dry ice and stored at −80°C until further use. When present, worms were located in the earwig's body cavity. After being removed from their insect host, they were thoroughly cleaned by repeated rinsing with PBS solution to remove contaminants of host origin prior to being put in frozen storage. Categorization of the samples was done by measuring the largest nematode found in each earwig. UI earwigs have no detectable nematode infection. EI earwigs and early maturation nematodes correspond to an earwig infected by a nematode smaller than 2 mm. LI earwigs and late maturation nematodes correspond to an earwig infected by a nematode between 7 and 9 mm in length. For DM, samples were obtained by observing earwigs inside their rearing container with a water pool (6 cm diameter and 0.5–1 cm depth) in darkness for 5–6 h using a red light. Once a nematode was seen coming out of an earwig in water, both specimens were individually snap-frozen in liquid N_2_. All samples (*N* = 3 or 4 per stage, see figure 1) were collected between January and July 2020, except for one sample in the EI which was collected the following year from the same field population following the same sampling procedure. One LI sample was infected with multiple nematodes, all between 7 and 9 mm long, and only the largest nematode from this sample was processed for sequencing. Earwigs were sexed based on their morphological characteristics; however, nematodes were not, owing to technical difficulties.

### (c) Nucleotide extraction

Total RNA extraction was carried out using the Direct-zol RNA MicroPrep Kit (Zymo Research, USA) with an on-filter DNase treatment following the manufacturer's instructions. Samples were ground in 300 µl 1× PBS buffer with blue sterile tips in a 2 ml Eppendorf tube kept half dipped inside a mortar filled with liquid N_2_ to prepare for extraction. For earwigs, extractions were performed from their head (after removing the black eye pigments on the outer eye) to get the whole CNS, while for nematodes extractions were performed from their whole body. Quality and quantity of the extracted nucleotides were measured on a Qubit 2.0 Fluorometer (Life Technologies, USA) and NanoDrop. High-quality RNA samples were stored at −80°C until use.

### (d) RNA sequencing

RNA integrity was evaluated on a Fragment Analyzer (Advanced Analytical Technologies, USA), and total RNA sequencing library was prepared with the Zymo-Seq RiboFree Total RNA Library Kit (Zymo Research, USA). Prepared libraries were quality checked on a MiSeq and sequenced across two lanes of Illumina HiSeq 2500 V2 Rapid Sequencing with 100 bp paired-end (PE) reads at the Otago Genomics Facility, University of Otago, Dunedin, New Zealand.

### (e) Bioinformatics pipeline for RNA-seq data

The RNA-seq reads were quality checked with FastQC and trimmed with Trimmomatic v. 0.38 [32] with the following parameters: ILLUMINACLIP:TruSeq3-PE-2.fa:2:30:10 LEADING:3 TRAILING:3 SLIDINGWINDOW:4:20 MINLEN:36. Reads were then mapped to their respective draft genomes [33,34] using two-pass mapping with the option –sjdbOverhang set to 99 in STAR v. 2.7.9 [35]. RSEM v. 1.3.3 [36] was used to generate read counts per gene with the rsem-calculate-expression command.

### (f) Differential expression analysis and clustering

To understand how gene expression changed in both the earwig host and nematode parasite during infection, a differential expression analysis was carried out in R (v. 4.3.3) using the DESeq2 package [37]. The sample variation for expression profiles was assessed using principal component analysis (PCA) with regularized log-transformed data. For both earwig and nematode datasets, genes were retained if their expression count was >10 in at least three replicates. The DESeq2 analysis was modelled to regress out the batch effects and account for both the sex and time points (design: ∼ batch + sex + time points) for earwig and time points as main effect for nematode dataset (design: ∼ batch + time points). The pairwise differential expression was calculated between time points in both datasets using the Wald test. Genes were considered differentially expressed when the corrected *p*-value using the Benjamini–Hochberg method was below 0.05 and log_2_ fold change (FC) was more than or equal to 2. A non-duplicated list of all differentially expressed genes from each pairwise comparison was used for clustering analysis of expression patterns across time points using the DIANA algorithm in the DEGreport package (v. 1.38.5) [38] in R with a minimal cluster size of 20 genes.

### (g) Functional analysis

An over-representation analysis (ORA) was performed with gene ontology (GO) terms for the genes in each cluster to identify common functional roles. GO annotation was extracted from the genome annotation [33,34], and an org.db database was created for each species using the makeOrgPackage function from the AnnotationForge (v. 1.44.0) package. ORA was performed with enrichGO from the clusterProfiler package (v. 4.0) [39] with *q-*value cutoff of 0.05 and multiple correction with Benjamini–Hochberg with all other default parameters unless otherwise stated. For genes in clusters 3, 5 and 6, default parameters did not yield enriched terms, so we used more lenient thresholds with multiple corrections set to none and *q-*value cutoff of 1. The universal gene set included all the genes in the differential gene expression (DGE) analysis.

### (h) Motif analysis

Motif enrichment analyses were used to identify common transcription factor binding sites that may be involved in host manipulation. Analyses focused on the cluster of genes upregulated DM compared to UI, EI and LI stages. Motif enrichment analysis was carried out using the findMotifs.pl tool from the HOMER package (v. 5.1) [40] in FASTA mode. For the genes of interest, we took 500 bp on both sides of the transcription start site as foreground, and the same genomic window from all the expressed genes was used as the background.

## 3. Results

### (a) Differential gene expression analysis

**Figure 2.**
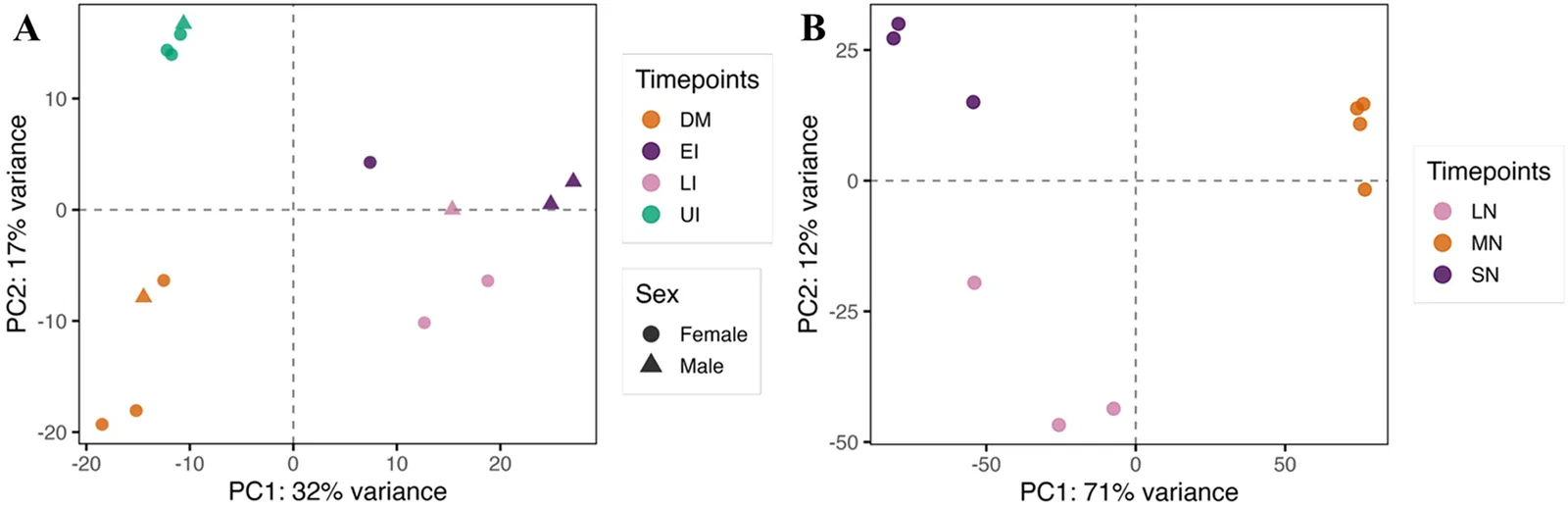
Principal component analysis (PCA) of the normalized RNA-seq data. (A) Earwig samples showing groupings based on infection status, with PC1 explaining 32% and PC2 17% of the variation. (B) Nematode samples coloured by their progressive developmental stages, as determined by size and change induced in behaviour, show grouping based on the time points, with PC1 explaining 71% and PC2 12% of the variance. Time points: for earwigs, UI = uninfected, EI = early-stage infection, LI = late-stage infection and DM = during manipulation; for nematodes, SN = early maturation, LN = late maturation and MN = manipulating worm.

A total of 213.1 million 100 bp PE reads were generated from earwig samples and 155.7 million 100 bp PE reads for nematode samples. PCA showed that the samples clustered primarily by infection or development stage for both earwigs and nematodes along PC1 and PC2, while no clustering was observed based on earwig sex (figure 2A,B). In earwigs, 32% of sample variation was explained by PC1 and 17% by PC2, whereas in nematodes, 71% of variation was explained by PC1 and 12% by PC2. Nematode samples, particularly those from the manipulation stages (MN), exhibited clear separation from other stages, while earwig samples formed three separate clusters: UI, EI and LI stages and DM.

**Figure 3.**
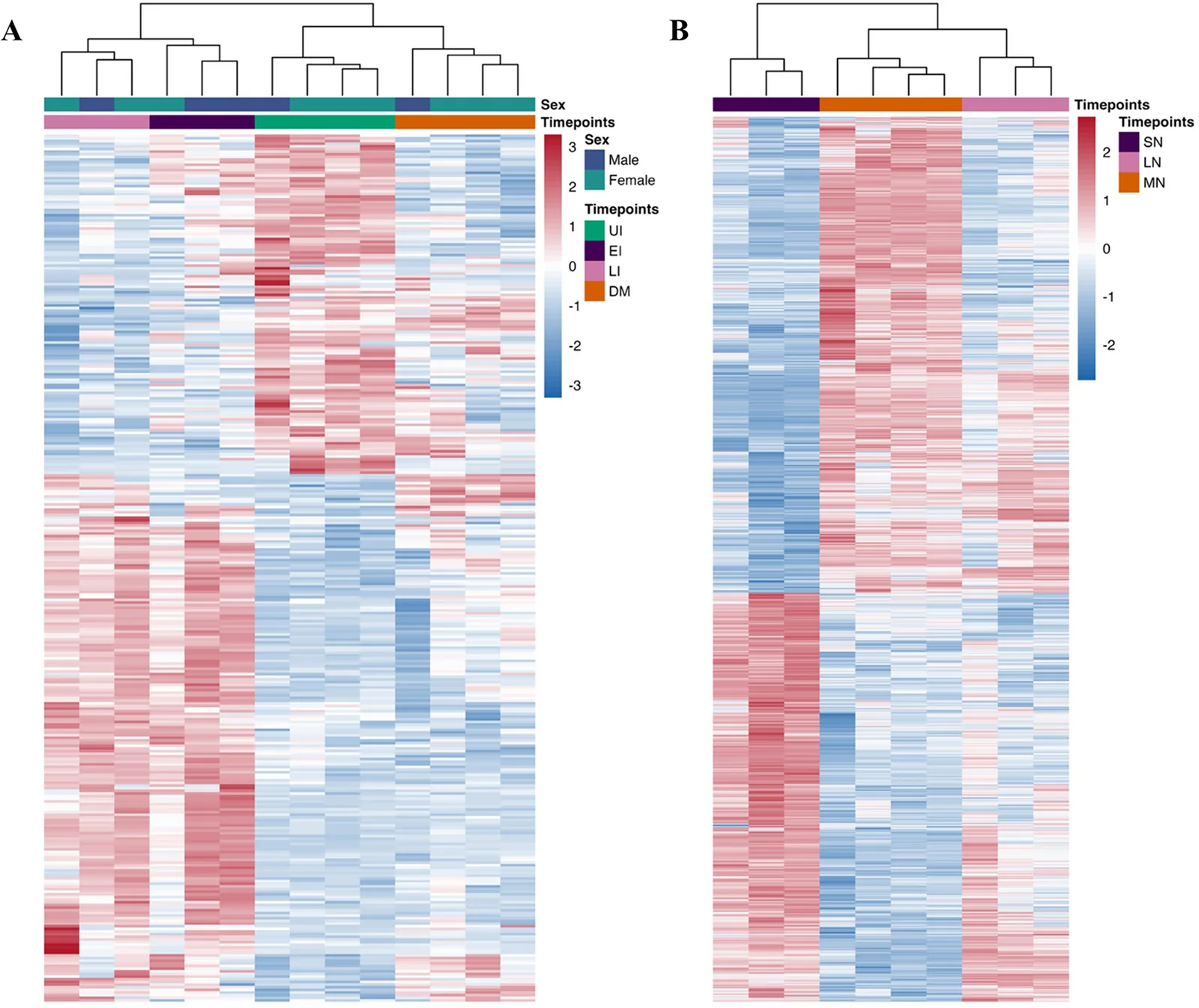
Heat map showing normalized, regularized logarithm (rlog)-transformed read counts of top 500 differentially expressed genes (*p*_adj._ < 0.05) for earwig samples (A) and nematode samples (B). Each row of the heat map represents a gene differentially expressed across all samples, colour-coded according to Wald statistic test scores, where red indicates upregulation and blue downregulation of a gene expression (gradient colour bar on the right). Each column represents an individual. Colours above the heat map indicate the sample categories as indicated by legends on the right. Time points: for earwigs, UI = uninfected, EI = early-stage infection, LI = late-stage infection and DM = during manipulation; for nematodes, SN = early maturation, LN = late maturation and MN = manipulating worm.

A total of 12 876 and 9722 genes were detected in earwig and nematode datasets, respectively. Among these, 673 genes were significantly upregulated and 593 genes downregulated in earwigs, and 2672 genes were upregulated and 2293 genes downregulated in nematodes across all pairwise comparisons (Wald tests, false discovery rate (FDR) < 0.05 and |log_2_ FC| ≥ 2). Heat maps revealed clear clustering of samples by developmental stages in both species (figure 3A,B).

Pairwise differential expression analysis in earwigs showed 34 upregulated and 93 downregulated genes between UI and EI, whereas only 1 gene was upregulated and 5 downregulated between EI and LI. Comparisons between LI and DM showed 187 upregulated and 46 downregulated genes. Similarly, 41 genes were up- and 75 were downregulated between UI and LI, 162 up- and 55 downregulated between UI and DM, and 256 up- and 75 downregulated between EI and DM.

In the nematode dataset, 181 genes were upregulated and 1111 genes downregulated between small nematode (SN) and large nematode (LN), 609 up- and 805 downregulated between LN and manipulating nematode (MN), and 761 up- and 1730 downregulated between SN and MN. Volcano plots illustrate the differences in magnitude and number of differentially expressed genes for each comparison (supplementary material, figure S1). In both species, the changes of gene expression heightened during the manipulation stage.

### (b) Clustering of differentially expressed genes based on expression patterns

**Figure 4.**
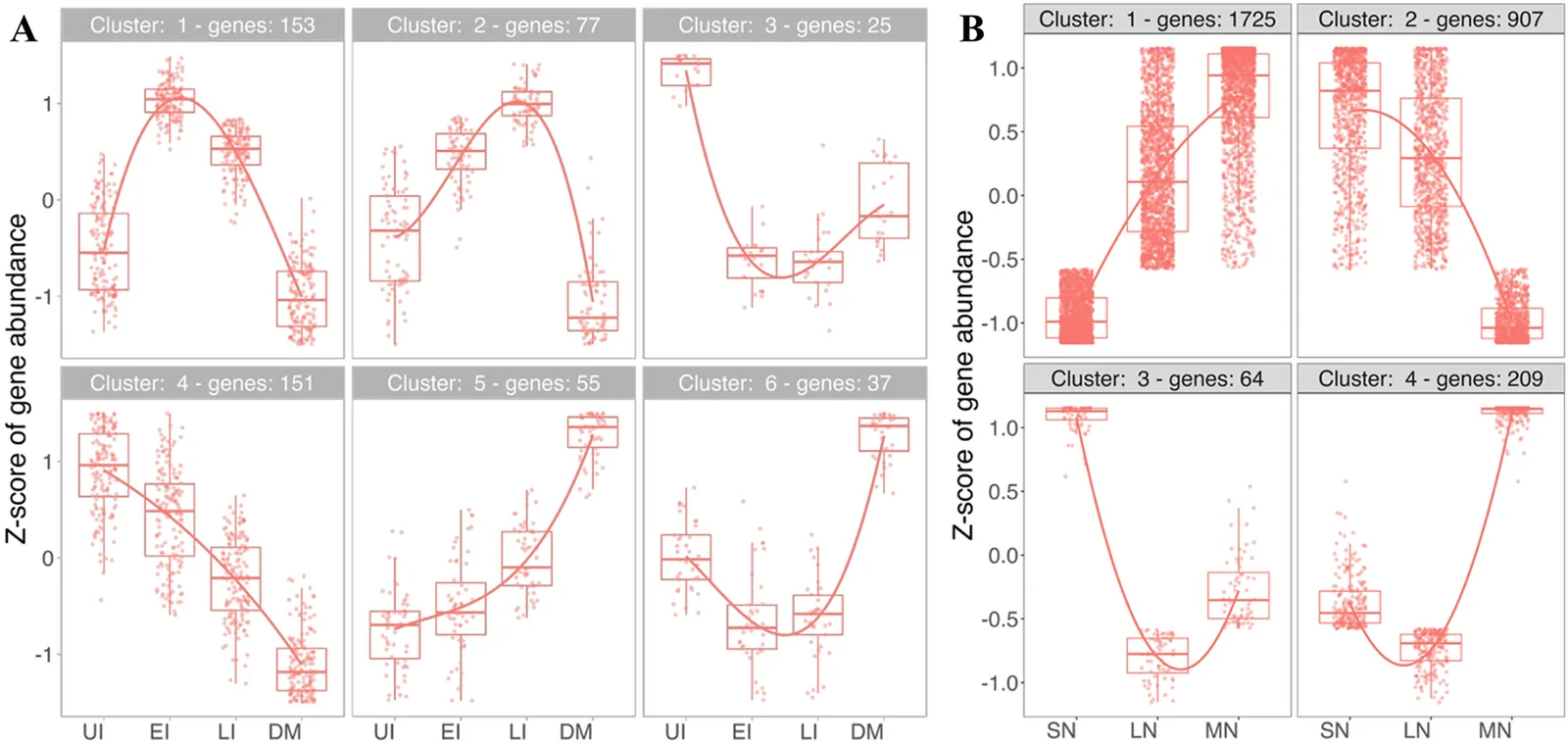
Cluster analysis of significantly expressed genes for earwig samples (A) and nematode samples (B). The *y*-axis shows the *Z*-score of gene abundance; the values are centred to the mean and scaled to the standard deviation for each gene. The *x*-axis shows different sampling stages. Each dot represents the expression of a gene; their relative expression values are summarized by box plots and whiskers at each sampling point. The curved lines represent the expressed trends. The number of genes in each cluster is given at the top of each cluster. Time points: for earwigs, UI = uninfected, EI = early-stage infection, LI = late-stage infection and DM = during manipulation; for nematodes, SN = early maturation, LN = late maturation and MN = manipulating worm.

The heat maps of the differentially expressed genes (DEGs) revealed distinct gene groupings across the various time points in both the earwig and nematode datasets (figure 3). To identify genes likely associated with host manipulation rather than those associated with infection or general nematode development, DEGs were clustered based on their expression profiles across time points (figure 4A,B).

In the earwig dataset, six gene clusters with distinct patterns were identified. Clusters 1, 2 and 4 are characterized by reduced expression during the manipulation stage compared with earlier time points. In contrast, cluster 5 includes genes showing a steady increase in expression from UI earwigs through all stages of infection (EI and LI) and DM. Expression of the genes in cluster 3 reduced after infection to LI and recovered slightly at DM, whereas the genes in cluster 6 peaked at DM after a slight decrease from UI to EI and LI. Cluster sizes range from 25 to 153 genes, with cluster 1 containing 153 genes, cluster 2 with 77, cluster 3 with 25, cluster 4 with 151, cluster 5 with 55 and cluster 6 with 37 genes. Of particular interest are cluster 2, which shows a pronounced drop in expression at manipulation stage, and cluster 6 which shows decreased expression following infection but rises DM. These clusters may provide insights into the gene expression changes driving behavioural manipulation.

In the nematode dataset, DEG clustering yielded four groups with distinct temporal dynamics. Cluster 1 consists of genes that are consistently upregulated from the early maturation (SN) to the late maturation (LN) and manipulative (MN) stages, whereas cluster 2 shows the opposite pattern, with genes that are consistently downregulated across these stages. Clusters 3 and 4 are notable in that their genes exhibit decreased expression from SN to LN but then show a significant increase from LN to the manipulation stage (MN). The gene counts in these clusters are 1725 for cluster 1, 907 for cluster 2, 64 for cluster 3 and 209 for cluster 4. Since our primary focus is on gene changes occurring during the manipulation stage, clusters 3 and 4 with their marked upregulation at MN compared with the previous infection stage represent particularly promising groups for functional analysis.

### (c) Functional analysis of significantly expressed genes in the earwig host

An ORA of within-cluster gene GO terms was used to identify key functions associated with each of the clusters. Earwig cluster 1 had significant enrichment in three GO terms. The most prominent term was extracellular region (*p*_adj._ 1.48e−08), which included genes *cda1*, *obst-e*, *chit1*, *cht10* and *ForAur_00000656*. These same genes were also enriched for the chitin binding category (*p*_adj._ 2.82e−07). Additionally, the carbohydrate metabolic process term was enriched (*p*_adj._ 2.86e−03), with genes including *chit1*, *cht10*, *cda8*, *cda1*, *cht2* and *gusb*.

**Figure 5.**
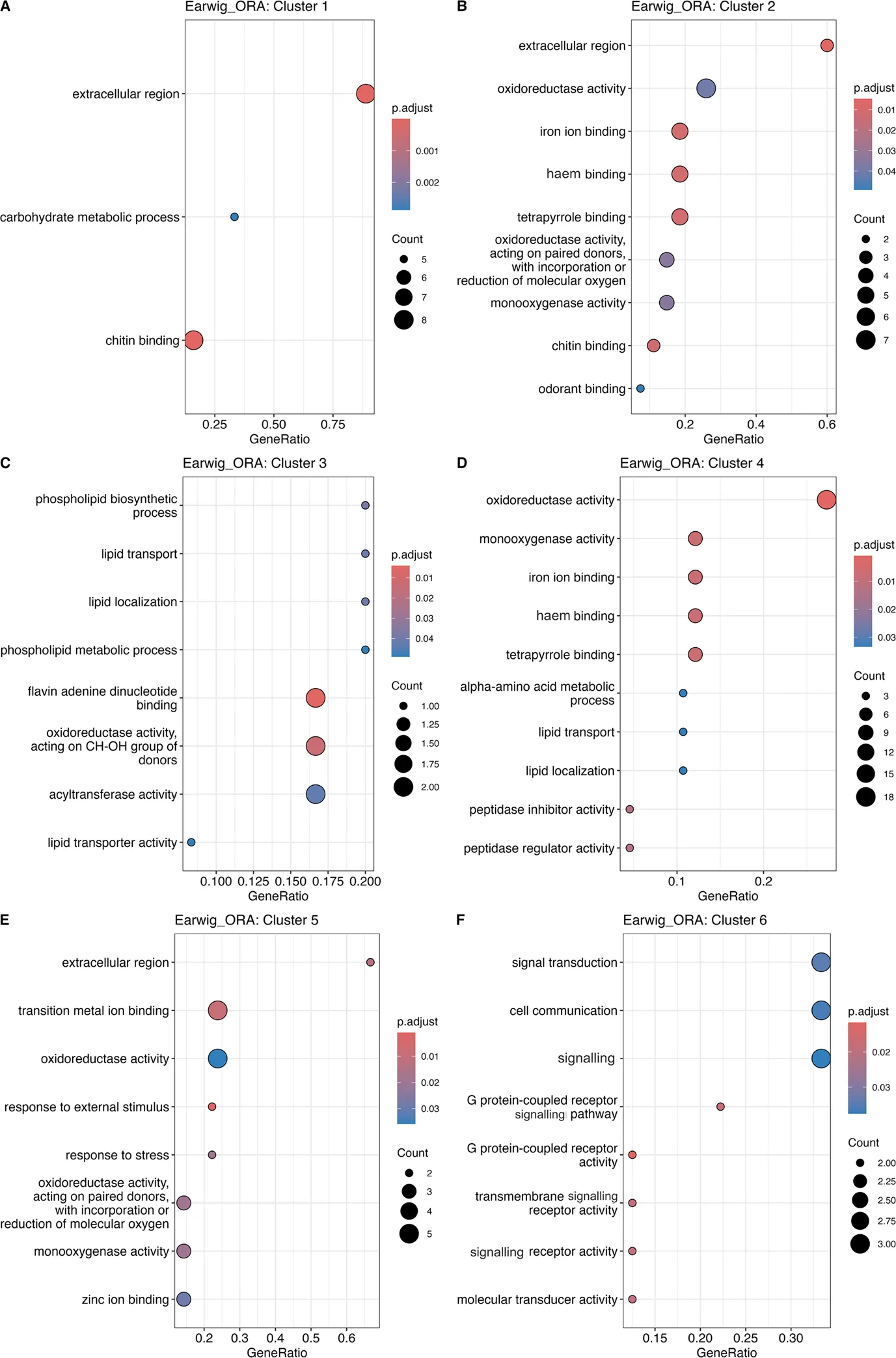
Dot plots showing top 10 enriched GO terms from the over-representation analysis of genes in earwig clusters 1 (A), 2 (B), 3 (C), 4 (D), 5 (E) and 6 (F) (see figure 4 for an explanation of clusters). Adjusted *p*-values are shown by the colour gradient, red to blue for low to high; the size of the dot indicates the gene count of each term. The *x*-axis represents gene ratio (scale varies between plots), and enriched terms are listed on the *y*-axis.

For cluster 2, extracellular region (*p*_adj._ 0.004) and chitin binding (*p*_adj._ 0.012), incorporating *cda1*, *chit1* and *obst-e* genes, were again enriched. Other terms enriched in this cluster include iron ion binding, haem binding and tetrapyrrole binding. There was notable overlap of genes across these terms, particularly *cyp15a1*, *cyp9e2*, *cyp4c1*, *agmo* and *obst-e* (figure 5B). Cluster 4 presented 15 enriched GO terms, with many enriched terms overlapping with cluster 2 (figure 5D).

Clusters exhibiting increased expression during the manipulation stage compared with EI and LI, namely clusters 3 and 5, initially did not yield significantly enriched terms under the *q-*value cutoff of <0.05 and Benjamini–Hochberg multiple correction. Using a more lenient threshold (*p*AdjustMethod = ‘none’, *q*valueCutoff = 1), cluster 3 returned eight enriched GO terms, four of which belonged to the biological process category. Each featured a gene ratio of 0.2, with *vg2* associated with lipid transport and localization and *hmgcs-1* involved in phospholipid biosynthetic and metabolic processes. The remaining four enriched terms were from the molecular function category, involving genes such as *gld*, *beta*, *vg2*, *nrf-6*, *oacyl* and *hmgcs-1* (figure 5C). Cluster 5 also showed increased expression throughout infection and manipulation stages. Enriched GO terms included extracellular region, transition metal ion binding, oxidoreductase activity, response to external stimuli, response to stress, and others (figure 5E).

**Figure 6.**
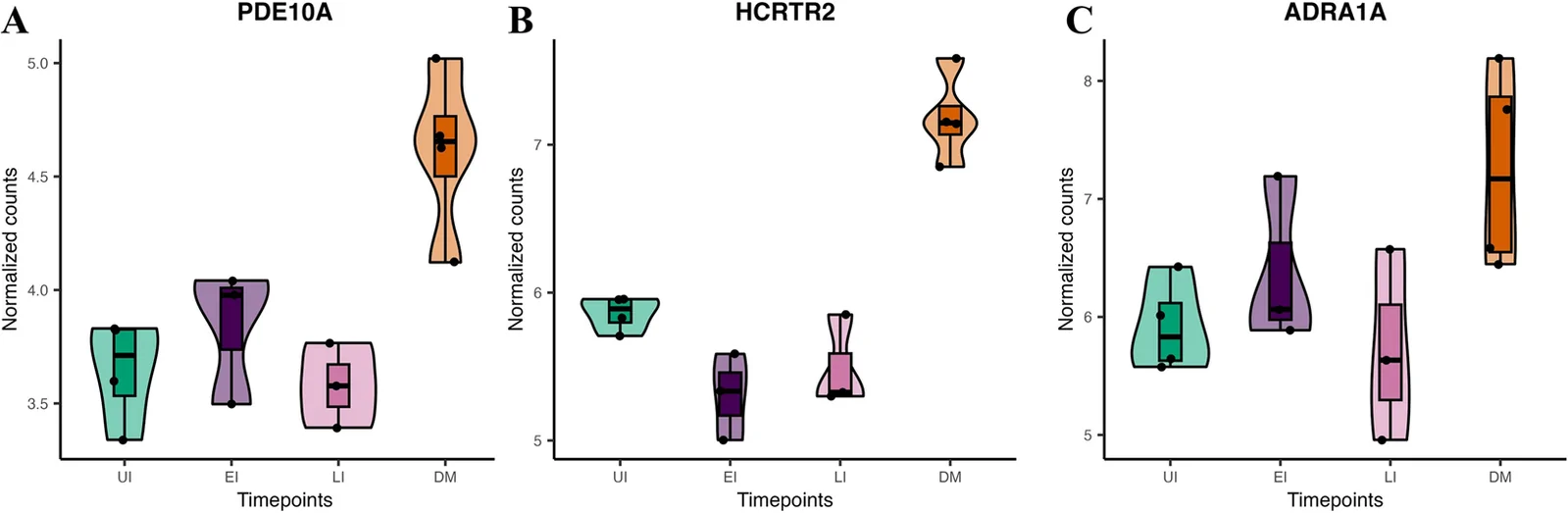
Expression of genes from enriched pathways in cluster 6 of the earwig dataset (see figure 4 for explanation of cluster 6). (A) *pde10a*, (B) *hcrtr2* and (C) *adra1a* gene expression. The *y* -axis represents log_2_-normalized gene counts, and the *x* -axis represents the infection stages of the earwig. UI = uninfected, EI = early infection stage, LI = late infection stage and DM = during manipulation.

The most intriguing findings emerged from cluster 6, where gene expression was high during the manipulation stage after being downregulated during EI and LI (compared with UI stage). Most of the enriched terms in this cluster are related to signalling, particularly transmembrane signalling. These included signal transduction, cell communication, G protein-coupled receptor (GPCR) activity and pathway, transmembrane signalling receptor activity and molecular transducer activity. Notably, just three genes, *hcrtr2*, *adra1a* and *pde10a*, were involved across all these enriched terms, and each exhibited higher expression during DM than at other time points (figure 6).

### (d) Motif analysis of earwig genes putatively involved in host manipulation

**Figure 7.**
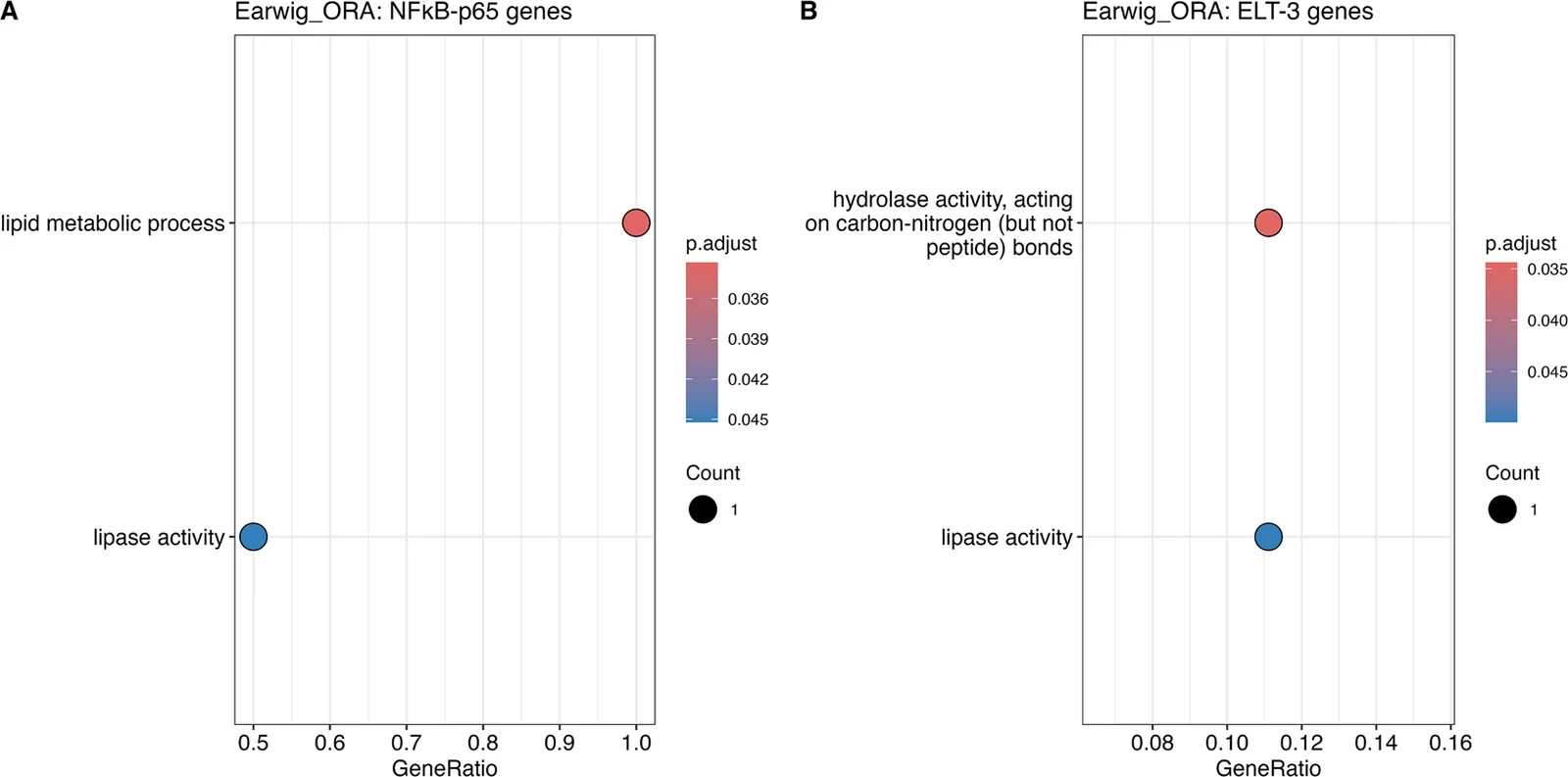
Dot plots showing enriched GO terms from the over-representation analysis of genes potentially linked to manipulation from two of the over-represented motifs. (A) Genes with NFκB-p65 motif enrichment and (B) genes with ELT-3 motif enrichment. Adjusted *p*-values are shown by the colour gradient, red to blue for low to high; the size of the dot indicates the gene count of each term. The *x*-axis represents gene ratio, and enriched terms are listed on the *y*-axis.

Motif analyses were used within clusters with gene expression changes DM to identify common gene regulatory regions. Enrichment of common motifs across candidate manipulation genes could suggest that the regulatory control of behavioural manipulations is linked to specific transcription factors. Motif analysis of genes in cluster 6 revealed nine known motifs (supplementary material, table S1). Several of these motifs, including NFκB-p65, NFkB-p50, Fra2 and TF3A, are known regulators of immune responses and inflammation [41–44]. Among them, NFκB-p65 was the most enriched motif associated with 11 genes. ORA of GO terms for this gene set indicated enrichment for lipid metabolic process (*p*_adj._ 0.03) and lipase activity (*p*_adj._ 0.04), each represented by a single gene (*lip3* and *plbd2*, respectively) (figure 7A).

The GATA transcription factor ELT-3, a known regulator of ageing in worms [45], is surprisingly one of the enriched motifs in cluster 6 genes in the earwig. ORA using a lenient threshold has earlier identified two enriched GO terms: hydrolase activity, acting on carbon–nitrogen bonds (*p*_adj._ 0.03), and lipase activity (0.04), each represented by one gene (*pgrp* and *plbd2*, respectively) (figure 7B).

### (e) Functional analysis of significantly expressed genes in the nematode

Clusters 1 and 2 in the nematode dataset showed distinct expression trends across time, with cluster 1 genes exhibiting progressively increased expression and cluster 2 genes showing a decrease from SN to MN. ORA identified 49 significantly enriched GO terms for cluster 1 and 23 for cluster 2. For cluster 1, top terms highlighted robust involvement in signalling and cellular communication, including pathways related to GPCR signalling and transmembrane transport (*p*_adj._ 9.8e−06), cell communication (*p*_adj._ 1.52e−05), signalling (*p*_adj._ 1.6e−05) and signal transduction (*p*_adj._ 4.33e−05). Additional terms pointed to heightened cellular responses to external stimuli (*p*_adj._ 2.41e−03). Together, these results suggest a pronounced increase in transmembrane signalling activity in this group.

**Figure 8.**
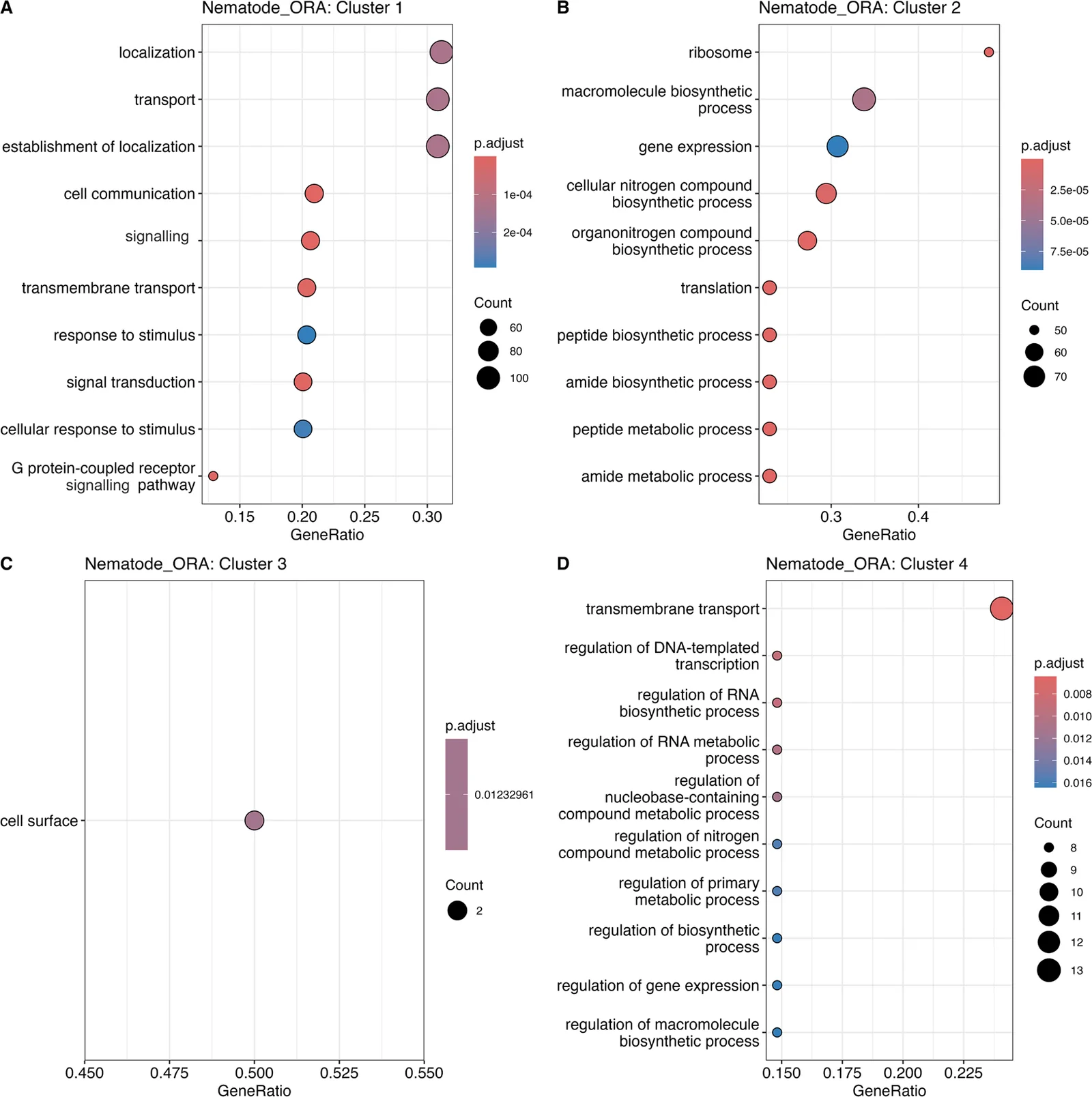
Dot plots showing the top 10 enriched GO terms from the over-representation analysis of genes in nematode clusters 1 (A), 2 (B), 3 (C) and 4 (D). Adjusted *p*-values are shown by the colour gradient, red to blue for low to high; the size of the dot indicates the gene count of each term. The *x* -axis represents gene ratio, and enriched terms are listed on the *y*-axis.

The most prominent features of cluster 2 were terms associated with biosynthetic and metabolic processes. The most significant terms included translation (*p*_adj._ 4.69e−19), and the following terms were peptide and amide biosynthetic and metabolic processes. This pattern indicates widespread downregulation of biosynthetic and metabolic activity in cluster 2 (figure 8).

Cluster 3, characterized by a modest increase in expression at the manipulation stage (MN) following a decrease from SN to LN, yielded a single significantly over-represented term, cell surface (*p*_adj._0.01), including the genes *ttr-52* and *ttr-2*. In cluster 4, where expression strongly increased at MN after a slight decline from SN to LN, the standard threshold did not yield significant enrichment. However, by relaxing the threshold as before, 25 enriched terms were identified. The most notable among these were transmembrane transport (*p*_adj._ 0.005), regulation of DNA-templated transcription and RNA biosynthetic processes (*p*_adj._ 0.014), and various metabolic and gene regulatory processes.

Within the transmembrane transport category, the majority of enriched genes encoded solute carrier proteins, such as *slc15a2*, *slc2a3*, *slc17a5*, *slc18a2* and *slc39a10*, as well as other transport-related proteins like *abcc3*, *oatp74d*, *pept-3*, *trpc2*, *kha1*, *orct* and *cac*. Many top enriched terms also consistently featured a set of regulatory genes, including *lrrfip2*, *meis3p2*, *ehf*, *foxp1*, *foxo2*, *st18*, *sgf1* and *eip74ef* (figure 8).

### (f) Motif analysis of nematode genes putatively involved in host manipulation

**Figure 9.**
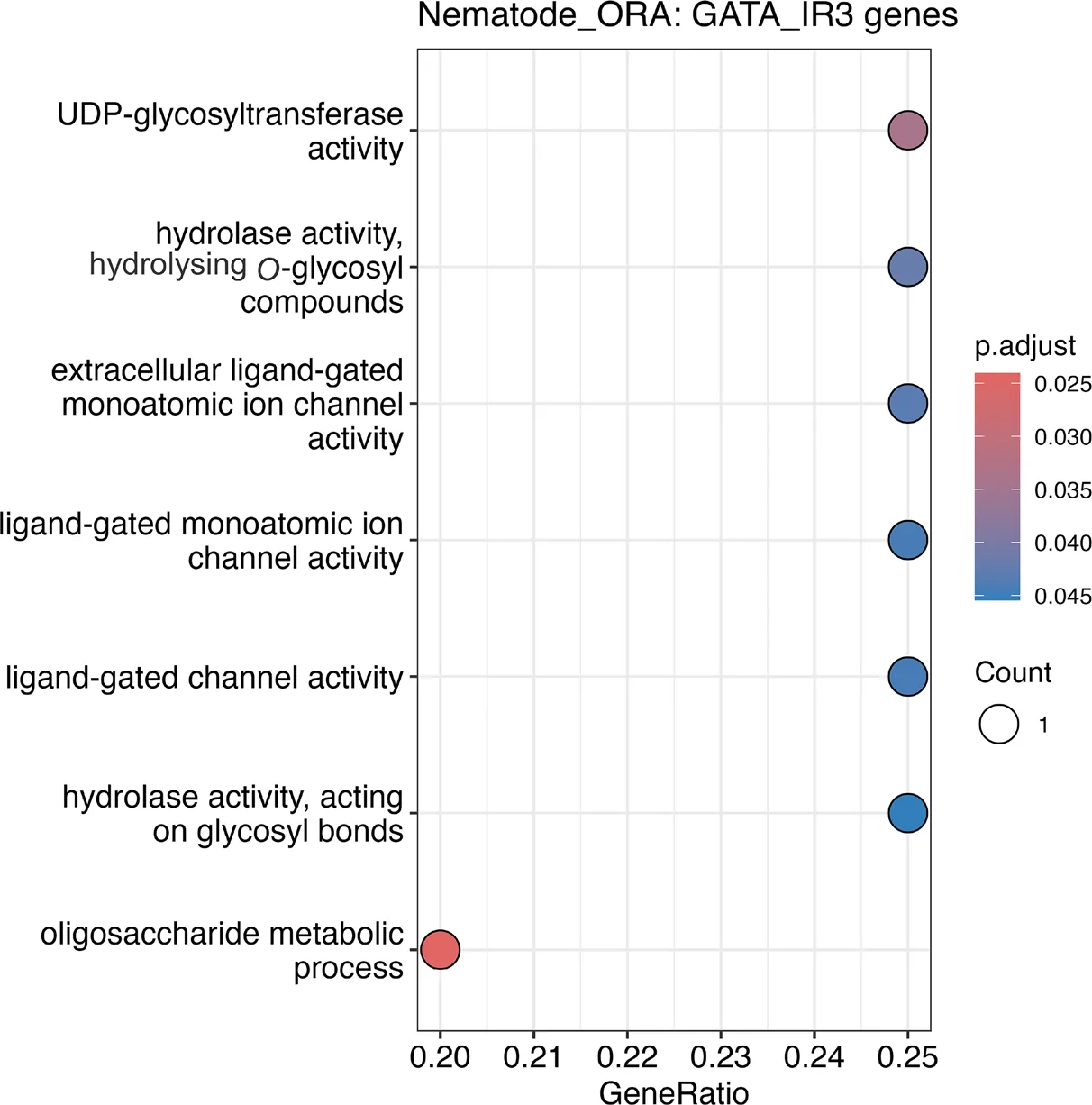
Dot plots showing enriched GO terms from the over-representation analysis of genes with GATA-IR3 motif enrichment. Adjusted *p*-values are shown by the colour gradient, red to blue for low to high; the size of the dot indicates the gene count of each term. The *x* -axis represents gene ratio, and enriched terms are listed on the *y*-axis.

Motif analysis of nematode cluster 4 genes identified six enriched known motifs: ABF1, GATA (Zf) IR3, AGL95 (ND), NFE2L2 (bZIP), Bach1 (bZIP) and PHA-4 (forkhead) (supplementary material, table S2). Among these, GATA-IR3, a zinc finger DNA-binding motif, though evolutionary distant, shares fundamental structural similarities with ELT-3, the GATA zinc finger motif enriched in earwig cluster 6 genes. ORA using this motif revealed seven enriched GO terms at a lenient threshold as earlier (figure 9), each represented by a single gene. These included: UDP-glycosyltransferase activity, extracellular ligand-gated monoatomic ion channel activity and oligosaccharide metabolic process-containing gene *tre1*; ligand-gated monoatomic ion channel activity, ligand-gated channel activity and hydrolase activity acting on glycosyl bond-containing gene *exp-1*; and hydrolase activity, hydrolysing *O*-glycosyl-compound-containing gene *ugt3*.

## 4. Discussion

We hypothesized that the nematode manipulates earwig behaviour by interfering with the host's CNS, thereby altering information processing and decision-making by the host [31]. In earwigs, EI versus LI produced relatively few DEGs. This limited change may reflect the nematode's growth inside the abdomen having only subtle effects on the CNS until the parasite reaches the manipulation stage. In contrast, nematode samples represented distinct developmental stages, which likely explains the larger number of DEGs observed between stages. This pattern supports the notion that nematodes remain ‘under the radar’ until they are fully developed and ready to emerge, an adaptive strategy critical for completing their life cycle.

Focusing on the genes differentially expressed DM, functional analysis revealed enriched signalling processes in both species. In earwigs, genes involved in transmembrane signalling were upregulated in the CNS, while nematodes showed increased activity of genes linked to carrier functions, molecular transport and regulatory processes. These complementary changes suggest a coordinated interaction in which parasite-driven transport and regulatory mechanisms may modulate host signalling, ultimately producing the behavioural shifts observed.

Keeping in mind that we used more lenient thresholds for enrichment and thus that the findings should be interpreted with caution, several host genes nevertheless emerged as particularly noteworthy. In cluster 6 of the earwig data, *hcrtr2*, *adra1a* and *pde10a* were strongly associated with enriched GO terms. The gene *pde10a* encodes phosphodiesterase 10A, a cyclic nucleotide phosphodiesterase involved in signal transduction and the dopamine receptor-associated second messenger pathway [46,47]. Its role in cyclic guanosine monophosphate (cGMP) hydrolysis is well established in both vertebrates and invertebrates [47,48], and loss of *pde10a* expression has been linked to reduced locomotion and cognitive defects in mice [49,50]. Notably, earwigs exhibit increased walking during the manipulative stage (personal observation), which may increase their chances of encountering water. This behavioural correlation highlights the *pde10a* gene as an interesting candidate gene for future investigation. The other genes, *adra1a*, encoding the adrenoceptor alpha 1A protein, and *hcrtr2*, encoding hypocretin receptor 2 proteins, are involved in GPCRs, a family of transmembrane proteins involved in diverse physiological and regulatory functions, including immune responses and sensory perception in insects [51,52]. Their expression further underscores the role of CNS signalling pathways in host manipulation.

On the parasite side, cluster 4 nematode genes were enriched for transmembrane transport and transcriptional and translational regulation. These processes are consistent with the secretion of molecules that could influence earwig signalling. Motif analysis revealed enrichment of conserved regulatory elements, including the zinc finger GATA motif in both species and NFκB-p65 as the most enriched in earwig. The lipid metabolism-related pathways were enriched in the earwig genes with those motifs. Lipid metabolism is tightly linked to neural function and cognitive processes [53]. One of the genes enriching lipase activities, *lip3*, is a regulator of metabolic responses to starvation and ageing [54]. The host's candidate genes with ELT-3 motifs enriching the functional GO terms are *pgrp* and *plbd2*. The *pgrp* gene encodes peptidoglycan recognition proteins (PGRPs); these are highly conserved pattern recognition proteins and are known to activate immune and neurobehavioural pathways [55,56].

In nematodes, GATA-IR3 motif-enriched genes include *tre1*, which is a GPCR, essential for germ cell migration in *Drosophila* [57]; similarly, *exp-1* is important for nutrient uptake in parasitic worms [58]. The *ugt3* (UDP-glucuronosyltransferase) gene is involved in detoxification and longevity [59] and may also contribute to the nematode's ability to survive within the host and influence host signalling.

Our findings are derived from naturally infected earwigs. We used worm size as a proxy for developmental stage, with no information on how ‘old’ the infection was. An obvious next step would be to conduct a similar study with experimentally infected hosts, in order to control the timing and dose of infection and reduce inherent variability among individual hosts used in the study. Nevertheless, taken together, our findings reveal that both earwigs and nematodes undergo coordinated transcriptional changes during the manipulation stage. It is possible that some of these changes serve a different function, that is, preparing the nematode for the physiological adjustments required for its free-living adult stage in a water-saturated substrate. However, many of the transcriptional changes we observed seem aligned with a role in host manipulation. Host genes linked to CNS signalling and parasite genes involved in transport, secretion and regulation converge on shared functional pathways. The enrichment of conserved *cis*-regulatory motifs across both species suggests common molecular switches that may mediate cross-species communication. This interplay between host signalling and parasite regulation provides a molecular framework for understanding the remarkable behavioural manipulation observed in this system and highlights candidate genes for future functional validation.

## Supplementary Material



## Data Availability

Raw sequence reads for both the host and the parasites are deposited in the SRA repository under the BioProject PRJNA1364760. Supplementary material is available online [60].
