# Supplementary material for: Revealing the genetic mechanisms underpinning the parasite-induced water-seeking behaviour of insects through RNA-seq
Source: Proc Biol Sci. 2026 Sep 16;293(2079):20261414. doi: 10.1098/rspb.2026.1414 (PMC13577805; doi:10.1098/rspb.2026.1414)
Supplement: Supplementary file 1 [file rspb.2026.1414.zip › rspb20261414_si_001.docx]

**SUPPLEMENTARY MATERIALS**

**Revealing the genetic mechanisms underpinning the parasite-induced water-seeking behaviour of insects through RNA-seq**

Upendra R. Bhattarai^1,2^, Jean-François Doherty^3,4^, Robert Poulin^5^, Eddy Dowle^6^, Neil J. Gemmell^1*^

^1^ Department of Anatomy, University of Otago, Dunedin, New Zealand

^2^ Department of Molecular Biology, Cell Biology, and Biochemistry, Brown University, Providence, RI, USA

^3^ Department of Zoology, The University of British Columbia, Vancouver, BC, Canada

^4^ Michael Smith Laboratories, Department of Biochemistry & Molecular Biology, The University of British Columbia, Vancouver, BC, Canada

^5^ Department of Zoology, University of Otago, Dunedin, New Zealand

^6^ New Zealand Institute for Bioeconomy Science Limited, Lincoln, Christchurch, New Zealand

*Corresponding author: Neil J. Gemmell (neil.gemmell@otago.ac.nz)

Figure S1. Volcano plots of differentially expressed genes in pairwise comparisons. Plots comparing earwig samples are in black outline and those comparing nematodes are in red. Comparison groups are indicated on top of each plot. In earwig samples UI=uninfected, EI=early infection, LI=late infection, DM=during manipulation timepoints, similarly, SN=small nematodes, LN=large nematodes, and MN=manipulating nematodes. Yellow dots denote genes with FDR <0.05 and log2FC ≥2. Blue dots denote genes with FDR <0.05 and log2FC <2. Purple dots represent genes with FDR >0.05 and log2FC ≥2. Grey dots represent genes with FDR >0.05, log2FC <2. There are 9,007 earwig genes, and 9,722 nematode genes in their respective analysis.

Table S1. Known motif enrichment in genes from cluster 6 of the earwig dataset (see Figure 4 for explanation of cluster 6).

| Motif name | Enriched motifs | p-values | Target sequences with motifs | Background sequences with motifs |
| --- | --- | --- | --- | --- |
| NFkB-p65 (RHD) |  | 1e-2 | 29.73% | 10.92% |
| TF3A (C2H2) |  | 1e-2 | 48.65% | 25.38% |
| NFkB-p50 , p52 (RHD) |  | 1e-2 | 10.81% | 1.64% |
| ELT-3 (GATA) |  | 1e-2 | 70.27% | 46.69% |
| HLF (bZIP) |  | 1e-2 | 86.49% | 66.05% |
| Fra2 (bZIP) |  | 1e-2 | 29.73% | 12.86% |
| CEBP:AP1 (bZIP) |  | 1e-2 | 56.76% | 35.27% |
| PLT1 (AP2EREBP) |  | 1e-2 | 13.51% | 3.38% |
| At1g19210 (AP2EREBP) |  | 1e-2 | 56.76% | 36.21% |

Table S2. Known motif enrichment in genes from cluster 4 of the nematode dataset (see Figure 4 for explanation of cluster 4).

| Motif name | Enriched motifs | p-values | Target sequences with motifs | Background sequences with motifs |
| --- | --- | --- | --- | --- |
| AB1F1 |  | 1e-3 | 35.41% | 25.26% |
| GATA (Zf), IR3 |  | 1e-2 | 11.96% | 6.36% |
| AGL95( ND) |  | 1e-2 | 8.13% | 3.94% |
| NFE2L2 (bZIP) |  | 1e-2 | 3.83% | 1.23% |
| Bach1 (bZIP) |  | 1e-2 | 3.83% | 1.32% |
| PHA-4 (Forkhead) |  | 1e-2 | 96.65% | 92.37% |
